# Potential of Establishing the Universal Critical Nitrogen Dilution Curve for Japonica Rice

**DOI:** 10.34133/plantphenomics.0036

**Published:** 2023-03-27

**Authors:** Zhaopeng Fu, Rui Zhang, Jiayi Zhang, Ke Zhang, Qiang Cao, Yongchao Tian, Yan Zhu, Weixing Cao, Xiaojun Liu

**Affiliations:** Sanya Institute, National Engineering and Technology Center for Information Agriculture, Key Laboratory for Crop System Analysis and Decision Making (Ministry of Agriculture and Rural Affairs), Engineering Research Center of Smart Agriculture (Ministry of Education), Jiangsu Key Laboratory for Information Agriculture, Jiangsu Collaborative Innovation Center for Modern Crop Production, Nanjing Agricultural University, Nanjing 210095, China.

## Abstract

Establishing the universal critical nitrogen (N_C_) dilution curve can assist in crop N diagnosis at the regional scale. This study conducted 10-year N fertilizer experiments in Yangtze River Reaches to establish universal N_C_ dilution curves for Japonica rice based on simple data-mixing (SDM), random forest algorithm (RFA), and Bayesian hierarchical model (BHM), respectively. Results showed that parameters *a* and *b* were affected by the genetic and environmental conditions. Based on RFA, highly related factors of *a* (plant height, specific leaf area at tillering end, and maximum dry matter weight during vegetative growth period) and *b* (accumulated growing degree days at tillering end, stem–leaf ratio at tillering end, and maximum leaf area index during vegetative growth period) were successfully applied to establish the universal curve. In addition, representative values (*most probable number* [*MPN*]) were selected from posterior distributions obtained by the BHM approach to explore universal parameters *a* and *b*. The universal curves established by SDM, RFA, and BHM-*MPN* were verified to have a strong N diagnostic capacity (N nutrition index validation *R*^2^ ≥ 0.81). In summary, compared with the SDM approach, RFA and BHM-*MPN* can greatly simplify the modeling process (e.g., defining N-limiting or non-N-limiting groups) while maintaining a good accuracy, which are more conducive to the application and promotion at the regional scale.

## Introduction

Rice (*Oryza sativa* L.) is the staple crop of and is widely cultivated in China [1]. Nitrogen (N) fertilizer can promote crop yield and affect the formation of final grain quality [2]. However, the N fertilizer overuse has a negative impact on crop production and environmental security [3]. Precise management of N fertilizer helps to prevent waste or shortage and effectively promotes crop growth and development [4,5].

Morphological comparison, chemical analysis, and instrumental detection are often used for determining the crop N nutrition [6–8]. The critical N (N_C_, %) dilution curve is considered as a reliable indicator in crop N status diagnosis [9,10]. Lemaire et al. [11] firstly proposed the concept of N_C_ dilution curve based on the relationship between plant N concentration and dry matter weight (DW, t ha^−1^). Plant N_C_ is defined as the minimum N concentration when DW reaches the maximum growing rate [12]. Under a certain biomass level, it is optimal for plant N concentration to reach the N_C_ value. Plant N concentration higher than the N_C_ value means that crop growth is not limited by N nutrition, while plant N concentration lower than the N_C_ value represents N-limiting status. In addition, the N nutrition index (NNI) was derived from the N_C_ dilution curve, which can effectively diagnose the N status of crops [13]. It is generally believed that an NNI value of 1 represents the appropriate state of crop N nutrition.

At present, some studies have established independent N_C_ dilution curves under specific cultivars, experiments, or other limitations. Parameters *a* and *b* varied between different N_C_ dilution curves due to cultivar and environmental condition differences, which increased the uncertainty [14–16]. In order to explore the variation sources of uncertainties, some scholars have focused on the uncertainty analysis of parameters *a* and *b* from crops’ N_C_ dilution curves, and results showed large differences between various species, cultivars, and field managements [17,18]. However, the traditional method of establishing N_C_ dilution curves by sampling and weighing measurements is time-consuming and laborious, and a new approach for rapid and accurate estimation of parameters *a* and *b* is urgently needed. Commonly used linear regression methods are difficult to deal with this kind of multi-factor problem. Meanwhile, machine learning methods are widely used in these issues because they can effectively weaken the collinearity influence between predictors [19,20].

In addition to parameter estimation of specific N_C_ curves, determining a universal N_C_ dilution curve is more practical for regional scale. Some works have been carried out to establish universal N_C_ dilution curves and realize the application for specific areas. Greenwood et al. [21] attempted to construct an N_C_ dilution curve for C3 plants, while limited modeling data could not accurately represent all the C3 crops. As for rice, relationships between plant N concentration and aboveground biomass varies under different cultivars, especially Japonica and Indica [22]. Therefore, it is worth exploring to establish the universal N_C_ dilution curve by setting partial restriction conditions (e.g., similar climate, cultivar, and cultivation region). For the regional scale, the universal N_C_ dilution curve is usually represented by simple data-mixing (SDM) of fitting all the N_C_ points determined by defining N-limiting and non-N-limiting groups and the linear-plateau model. Although this traditional approach has a good applicability, the process of determining all the N_C_ points is time-consuming. Moreover, many researchers adopted different growth parameters (e.g., leaf area index [LAI] and leaf dry matter) instead of the traditional plant DW, and the method of averaging the parameters from specific N_C_ dilution curves was verified in application and mathematical statistics [23–25]. These provide the possibility for calculating universal curve parameters according to estimated specific curve parameters. In addition to traditional simple linear regressions (e.g., multiple linear regression [MLR]), random forest algorithm (RFA) should also be considered for its high accuracy and wide application in crop parameter estimation. As a learning method integrating multiple decision trees, RFA can process large-scale information and reduce noise [26]. As another way, Makowski et al. [17] also proposed to adopt a Bayesian hierarchical model (BHM) approach to skip the step of distinguishing N-limiting or non-N-limiting groups. Combined with the prior knowledge, the posterior distribution of N_C_ dilution curve parameters can be obtained through the Markov Chain Monte Carlo (MCMC) method. As the main rice-producing area in China, Yangtze River Reaches is planted with plenty of Japonica rice cultivars [27]. Establishing the universal N_C_ dilution curve for Japonica rice will be of great significance to diagnose crops’ N status at the regional scale.

In this study, specific N_C_ dilution curves were established based on plant DW and N concentration in the vegetative growth period from numerous N fertilizer experimental data of Japonica rice in Yangtze River Reaches. Overall, the objectives were (a) to analyze the sources and uncertainties leading to differences in each specific N_C_ dilution curve; (b) to explore the potential of estimating parameters from specific N_C_ dilution curves using MLR and a machine learning method (RFA); and (3) to establish a universal N_C_ dilution curve for Japonica rice in Yangtze River Reaches based on SDM, RFA, and BHM approaches. These results will provide theoretical and technical support for field N precise management of Japonica rice.

## Materials and Methods

### Experimental design

Different N fertilizer rates (0 to 405 kg N ha^−1^) were examined across 10 years at 7 sites in Jiangsu Province, China: Yizheng (YZ) city in the growing seasons of 2010 to 2011, Wujiang (WJ) city in the seasons of 2013, Rugao (RG) city in the seasons of 2013 to 2014, Zhangjiagang (ZJG) city in the seasons of 2014, Sihong (SH) city in the seasons of 2015 to 2016, Huai’an (HA) city in the seasons of 2015, and Xinghua (XH) city in the seasons of 2017 to 2020. All experiments are composed of 10 japonica cultivars, including Lingxiangyou 18 (LXY 18), Wuxiangjing 14 (WXJ 14), Wuyunjing 19 (WYJ 19), Yongyou 8 (YY 8), Ningjing 4 (NJ 4), Lianjing 7 (LJ 7), Wuyunjing 24 (WYJ 24), Nanjing 9108 (NJ 9108), Yongyou 2640 (YY 2640), and Wuyunjing 32 (WYJ 32). In addition, experiments were also divided into 5 different crop management groups according to N application rates, fertilization ratio arrangement, and sampling frequency (A group: Exp. 1 and 2; B group: Exp. 3, 6, and 7; C group: Exp. 4 and 5; D group: Exp. 8 and 9; and E group: Exp. 10, 11, 12, and 13). Other specific information is presented in Table 1.

**Table 1.** Basic information about the experimental design. The regions of YZ, WJ, RG, ZJG, HA, SH, and XH represent Yizheng, Wujiang, Rugao, Zhangjiagang, Huai’an, Sihong, and Xinghua, respectively; the cultivars of LXY 18, WXJ 14, WYJ 19, YY 8, NJ 4, LJ 7, WYJ 24, NJ 9108, YY 2640, and WYJ 32 stand for Lingxiangyou 18, Wuxiangjing 14, Wuyunjing 19, Yongyou 8, Ningjing 4, Lianjing 7, Wuyunjing 24, Nanjing 9108, Yongyou 2640, and Wuyunjing 32, respectively. PP, TI, SE, PI, JT, BT, and HD represent the rice growth stages of Pre planting, Tillering, Stem elongation, Panicle initiation, Jointing, Booting, and Heading, respectively.

Experiment	Year	Region	Cultivar	N rates (kg N ha^−1^)	N fertilization ratio and date	Sampling stage and date
**Exp. 1**	2010	YZ	LXY 18, WXJ 14	0, 80, 160, 240, 320	PP (Jun 19):TI (Jul 04):JT (Jul 31):BT (Aug 12) = 5:1:2:2	TI (Jul 17), SE (Jul 26), PI (Aug 08), BT (Aug 20), HD (Aug 30)
**Exp. 2**	2011	YZ	LXY 18, WXJ 14	0, 90, 180, 270, 360	PP (Jun 20):TI (Jul 09):JT (Aug 01):BT (Aug 14) = 5:1:2:2	TI (Jul 21), SE (Jul 26), PI (Aug 02), BT (Aug 25), HD (Sep 03)
**Exp. 3**	2013	WJ	WYJ 19, YY 8	0, 90, 180, 270, 360	PP (Jun 18):TI (Jun 28):JT (Aug 12):BT (Aug 19) = 4:1:3:2	TI (Jul 18), SE (Jul 31), PI (Aug 13), BT (Aug 20)
**Exp. 4**	2013	RG	WXJ 14	0, 75, 150, 225, 300, 375	PP (Jun 19):TI (Jun 29):JT (Aug 03):BT (Aug 19) = 4:1:2:3	TI (Jul 19), SE (Jul 27), PI (Aug 04), BT (Aug 19)
**Exp. 5**	2014	RG	WYJ 24	0, 150, 225, 300, 375	PP (Jun 18):TI (Jul 02):JT (Aug 05):BT (Aug 22) = 4:1:2:3	TI (Jul 18), SE (Jul 30), PI (Aug 06), BT (Aug 16), HD (Aug 26)
**Exp. 6**	2014	ZJG	WYJ 19, YY 8	0, 90, 180, 270, 360	PP (Jun 6):TI (Jul 03):JT (Aug 04):BT (Aug 12) = 4:1:3:2	TI (Jul 23), SE (Aug 01), BT (Aug 18), HD (Aug 29)
**Exp. 7**	2015	HA	NJ 4, LJ 7, WYJ 24	0, 120, 240, 360	PP (Jun 12):TI (Jul 09):JT (Aug 14):BT (Aug 19) = 4:1:3:2	TI (Jul 25), SE (Aug 07), PI (Aug 14), BT (Aug 21), HD (Aug 28)
**Exp. 8**	2015	SH	NJ 4, LJ 7, WYJ 24	0, 120, 240, 360	PP (Jun 12):TI (Jul 07):JT (Aug 09):BT (Aug 12) = 3:2:3:2	TI (Jul 23), SE (Jul 30), PI, Aug 05; Booting, Aug 12; Heading, Aug 19
**Exp. 9**	2016	SH	NJ 4, LJ 7, WYJ 24	0, 120, 240, 360	PP (Jun 25):TI (Jul 06):JT (Aug 11):BT (Aug 16) = 3:2:3:2	TI (Jul 22), SE (Jul 28), PI (Aug 11), BT (Aug 18), HD (Aug 24)
**Exp. 10**	2017	XH	NJ 9108, YY 2640	0, 135, 270, 405	PP (Jun 14):TI (Jun 24):JT (Aug 06):BT (Aug 16) = 3:3:2:2	TI (Jul 16), SE (Jul 25), PI (Jul 31), BT (Aug 10), HD (Aug 23)
**Exp. 11**	2018	XH	NJ 9108, YY 2640	0, 135, 270, 405	PP (Jun 18):TI (Jun 28):JT (Aug 09):BT (Aug 20) = 3:3:2:2	TI (Jul 22), SE (Jul 28), PI (Aug 12), BT (Aug 19), HD (Aug 31)
**Exp. 12**	2019	XH	NJ 9108, YY 2640, WYJ 32	0, 135, 270, 405	PP (Jun 13):TI (Jun 23):JT (Aug 04):BT (Aug 15) = 3:3:2:2	TI (Jul 20), SE (Jul 27), PI (Aug 05), BT (Aug 15), HD (Aug 23)
**Exp. 13**	2020	XH	NJ 9108, YY 2640, WYJ 32	0, 135, 270, 405	PP (Jun 20):TI (Jun 30):JT (Jul 28):BT (Aug 25) = 3:3:2:2	TI (Jul 21), SE (Aug 03), PI (Aug 12), BT (Aug 23), HD (Sep 03)

### Data acquisition

At least 15 fresh rice plants were selected at critical growth stages from each plot in 13 experiments and separated by organs (leaf, stem, and spike). A portable li-3000c leaf area meter (Li-Cor., Lincoln, USA) was used for obtaining leaf area, and the LAI was calculated in Eq. 1 [28]. All samples were first heated for 30 min at 105 °*C* in a forced-draft oven, and then dried at 80 °*C* to a constant weight. Finally, dry matter weight of samples was weighed by a micrometer balance.

Plant DW includes DW of leaves, stems, and spikes, respectively. The specific formula of organ DW is as follows:

$$\mathrm{LAI}=\frac{r\times s\times 10^{-4}}{k}$$ (1)

$$\mathrm{DW}\;\left(t\;{\mathrm{ha}}^{-1}\right)=\frac{w\times s\times 10^{-2}}{k}$$ (2)

where *r* (cm^2^) represents the readings of the li-3000c leaf area meter, *w* (g) stands for the dry matter weight of samples (leaf, stem, or spike), *s* represents tiller number in 1 m^2^ from each plot, and *k* denotes the tiller number of sampled rice plants.

Stem–leaf ratio (SLR) represents the distribution of plant dry matter; it can be calculated as follows:

$$\mathrm{SLR}=\frac{\mathrm{SDW}}{\mathrm{LDW}}$$ (3)

in which SDW (g) and LDW (g) are the dry matter weight of stems and leaves, respectively.

The dry matter of each organ was ground into powder by a grinder and passed through a 1-mm sieve. Each sample was weighed to 0.15 g and N concentration (N%DW) was determined in a continuous-flow auto-analyzer (BRAN + LUEBEE, Hamburg, Germany) after Kjeldahl digestion and an isochoric process.

Accumulated growing degree days (AGDD, °C) is the cumulative value of growing degree days (GDD, °C). GDD refers to the cumulative effective accumulated temperature experienced by completing a certain growth stage under actual environmental conditions. They are calculated in Eqs. 4 and 5:

$$\mathrm{GDD}\;\left({}^{\circ}\;C\right)=\frac{T_{\mathrm{MAX}}-T_{\mathrm{MIN}}}{2}-T_{B}$$ (4)

$$\text{AGDD}\;\left({}^{\circ}\;C\right)=\sum_{k=1}^{n}\mathrm{GDD}$$ (5)

in which T*_MAX_* (°*C*) and T*_MIN_* (°*C*) are the maximum and minimum temperature in a day, respectively, T_B_ (°*C*) is the base point temperature of crop development (12.5 °*C* for Japonica rice) [29], and k is the number of days after rice transplanting.

Rice plant DW reaches 1 t ha^−1^ at about the end of tillering, and the plant N concentration at this time represents the value of parameter a. Therefore, the end of tillering was closely related to parameters a and b and was set as a time point to construct the indicators. A total of 14 factors were selected for parameter estimation, where thousand grain weight (TGW, g), amylose content (AC, %), plant height (PH, cm), specific leaf area at tillering end (SLA-T), and SLA at vegetative growth end (SLA-V) stood for the cultivar characteristics of Japonica rice; AGDD over the whole growth period (AGDD), AGDD at tillering end (AGDD-T), and AGDD during the vegetative growth period (AGDD-V) reflected the differences of growth days and temperature caused by years and regions; and maximum DW at the tillering stage (DW-T), maximum LAI during the tillering stage (LAI-T), SLR at tillering end (SLR-T), maximum DW during the vegetative growth period (DW-V), maximum LAI during the vegetative growth period (LAI-V), and SLR at vegetative growth end (SLR-V) indicated the impact of various influencing factors (cultivar, region, year, and crop management) on rice growth. TGW, AC, and PH were reference values of rice cultivar parameters obtained from the China Rice Data Center (https://www.ricedata.cn). They were used to represent the inherent potential of the rice cultivar, and do not mean that the measured value will not change due to cultivation environment. However, there were still great uncertainties in the application of these empirical indicators. SLA is the ratio of one-side leaf area to the corresponding DW [30], which is also an indicator of cultivar parameters in many crop growth models [31,32]. AGDD was calculated from the meteorological information of the local station. In addition, DW, LAI, and SLR were typical growth indicators measured through experimental tests [4,33].

### Approaches for establishing the universal N_C_ dilution curve

The following 3 approaches were selected to establish the universal N_C_ dilution curve in this study:

a. The SDM approach (i) defined non-N-limiting and N-limiting groups through variance analysis, (ii) determined the N_C_ point based on the linear-plateau model, and (iii) fitted all the N_C_ points to a power function curve.

b. The RFA approach (i) predicted parameters *a* and *b* of specific N_C_ dilution curves, and (ii) calculated parameters *a* and *b* of the universal N_C_ dilution curve by averaging the estimated parameters of specific curves. RFA takes the decision tree as the basic model and generates a series of decision tree models by constructing different training datasets and feature spaces. It is suitable for solving complex nonlinear problems [34]. NTREE (number of decision trees) and MTRY (number of observations per tree leaf) are critical tuning parameters in RFA. NTREE affected the operation time and was set to 500 as commonly used in previous studies [5]. The MTRY value had a great influence on the modeling accuracy, which was adjusted to achieve the smallest relative root mean square error (RRMSE) according to the input variables (Table 3). In addition, the RFA approach was compared with the MLR method, which is often used for dealing with multi-factor problems [35]. The 2 methods were both used for parameter estimation of specific N_C_ dilution curves, and validated by the leave-one-out cross-validation method. This validation method leaves only one sample as the test set and other samples as the training set. If there are *k* samples, *k* times training and testing are needed. It is suitable for a small number of samples and has high sample utilization [36].

c. The BHM approach (i) obtained the posterior distributions of parameters *a* and *b* based on the BHM and MCMC, and (ii) selected representative values (*Mean*, *Median,* and *most probable number* [*MPN*]) from the posterior distributions as universal curve parameters. The BHM was constructed based on the linear-plus-plateau of biomass to N concentration, which also considered the factors of measuring the date’s variation and prior knowledge, and the detailed process can be found in Makowski et al. [17]. MCMC, a parameter uncertainty analysis method based on Bayesian statistical theory, was adopted for estimating posterior distributions of universal N_C_ dilution curve parameters. The principle is to construct multiple stable Markov chains according to the parameter prior knowledge and model observation information, and explore the probability distribution of the parameters [24]. This method can be implemented by calling the rjags package in R Programming Language 3.6.0 software (R Foundation for Statistical Computing, Vienna, Austria). In this study, prior 1 (weakly informative priors) was refined without strong limitations (*a* ranged from 0 to 6, and *b* ranged from 0 to 1) for better estimation. The algorithm converged after about 50,000 iterations, and then additional 50,000 iterations were performed. Finally, posterior distributions were described by a frequency distribution histogram and kernel density function [37].

### Data processing and analysis

N_C_ dilution curve indicates that plant N concentration decreases with the increase of DW, which conforms to the form of power function:

$$N_{C}\;\left(\%\right)=a\times {\mathrm{DW}}^{-b}$$ (6)

in which *a* stands for plant N concentration when the biomass is 1 t ha^−1^, and *b* is the parameter affecting the slope of the curve. N concentration above the N_C_ dilution curve indicates excess N, N concentration on the curve means suitable N, and N concentration below the curve represents limited N.

Standard deviation (SD) and coefficient of variation (CV) were adopted to describe the basic attributes of the dataset, and the larger values of the two, the more scattered the data. They are calculated in Eqs. 7 and 8:

$$\mathrm{SD}=\sqrt{\frac{1}{n-1}\sum_{i=1}^{n}{\left(m_{i}-\bar{m}\right)}^{2}}$$ (7)

$$\mathrm{CV}=\frac{\mathrm{SD}}{\text{Mean}}$$ (8)

where $\bar{m}\;$ is the average value of total samples, and *n* stands for the number of samples.

Fluctuation range was introduced to represent the variation degree of parameters under different environmental conditions; the specific formula is as follows:

$$\text{Fluctuation range}=\frac{\text{Distribution range}}{\text{Mean}}$$ (9)

in which fluctuation range was calculated as the ratio of distribution range to mean value.

*R*^2^ and RRMSE were used to represent the accuracy and stability of models, respectively. The closer *R*^2^ was to 1 and the smaller RRMSE was, the better the model was. They are described in Eqs. 10 and 11:

$$R^{2}=\frac{\sum_{i=1}^{k}{\left(m-\bar{m}\right)}^{2}{\left(n-\bar{n}\right)}^{2}}{\sum_{i=1}^{k}{\left(m-\bar{m}\right)}^{2}\sum_{i=1}^{n}{\left(n-\bar{n}\right)}^{2}}$$ (10)

$$\mathrm{RRMSE}=\sqrt{\frac{1}{k}\sum_{i=1}^{k}{\left(m-n\right)}^{2}}/\bar{n}$$ (11)

in which *m* and *n* stand for the estimated values and measured values, respectively; $\bar{m}$ and $\bar{n}$ stand for the average estimated values and measured values, respectively; and *k* stands for the number of samples.

NNI is the ratio of measured N concentration to N_C_, which was used in this study to verify the N diagnostic capacity of the established universal N_C_ dilution curves. It can be calculated as:

$$\mathrm{NNI}=\frac{{\mathrm{NC}}_{T}}{N_{C}}$$ (12)

where *NC_T_* (%) represents the real-time measured N concentration and *N_C_* (%) is calculated by the N_C_ dilution curve. An NNI value greater than 1 indicates N surplus, an NNI value equal to 1 indicates N suitability, and an NNI value less than 1 indicates N deficiency.

Statistical analysis was performed using IBM SPSS 25 software (IBM Corporation, Armonk, USA). Significance analysis was completed in R Programming Language 3.6.1 (R Foundation for Statistical Computing, Vienna, Austria) by comparing the distributions of parameters.

### Technical flowchart

**Fig. 1.**
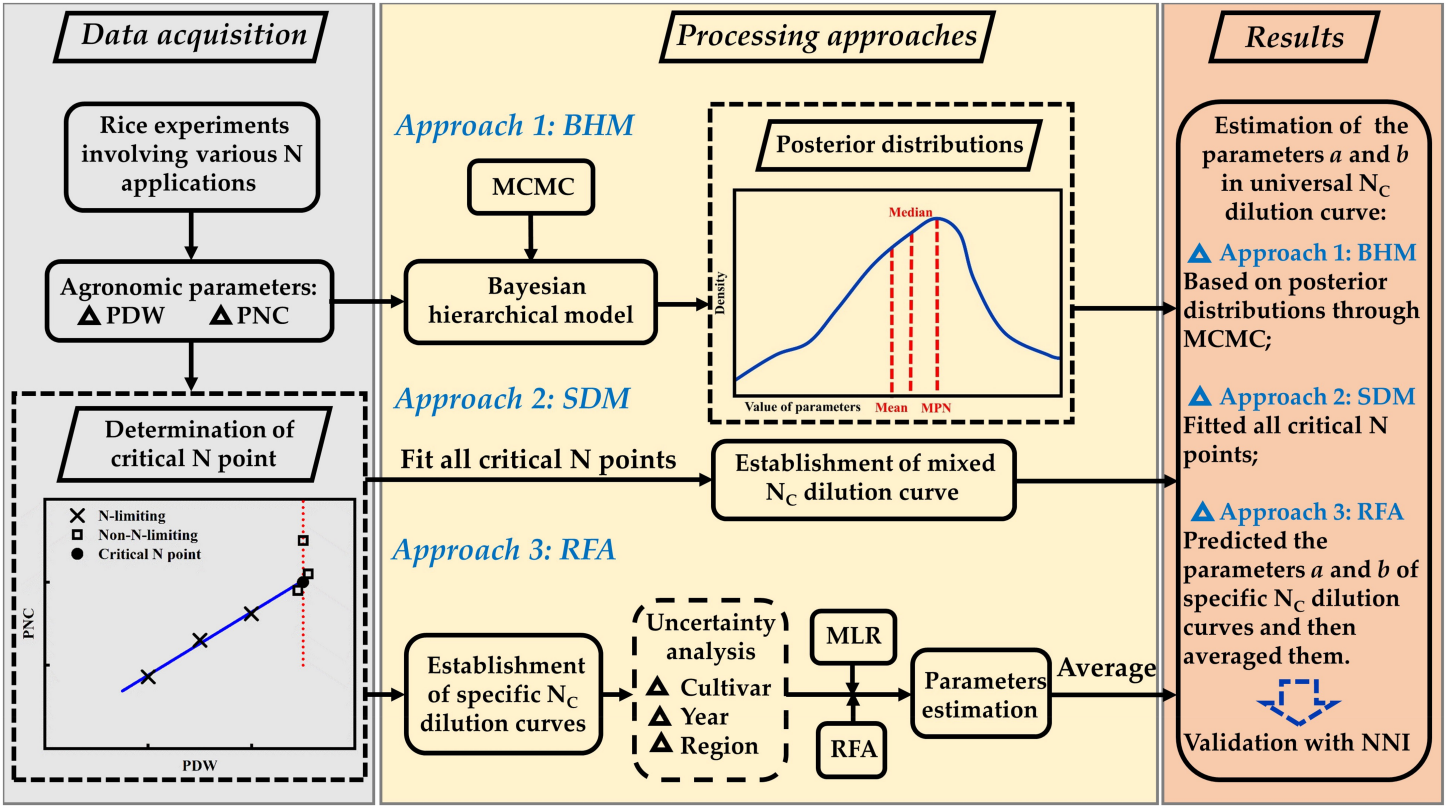
Technical flowchart of this study. BHM, Bayesian hierarchical model; SDM, simple data-mixing; RFA, random forest algorithm; PDW, plant dry matter weight; PNC, plant nitrogen concentration; MCMC, Markov Chain Monte Carlo; MLR, multiple linear regression; NNI, nitrogen nutrition index.

In this study, we established specific N_C_ dilution curves based on 10 years’ experiments in Yangtze River Reaches. Uncertainty factors affecting parameters *a* and *b* were analyzed from cultivar, year, region, and crop management. SDM, RFA, and BHM approaches were adopted to explore the establishment of a universal N_C_ dilution curve for Japonica rice. The whole technical flowchart is shown in Fig. 1.

## Results

### Establishment of specific N_C_ dilution curves

**Fig. 2.**
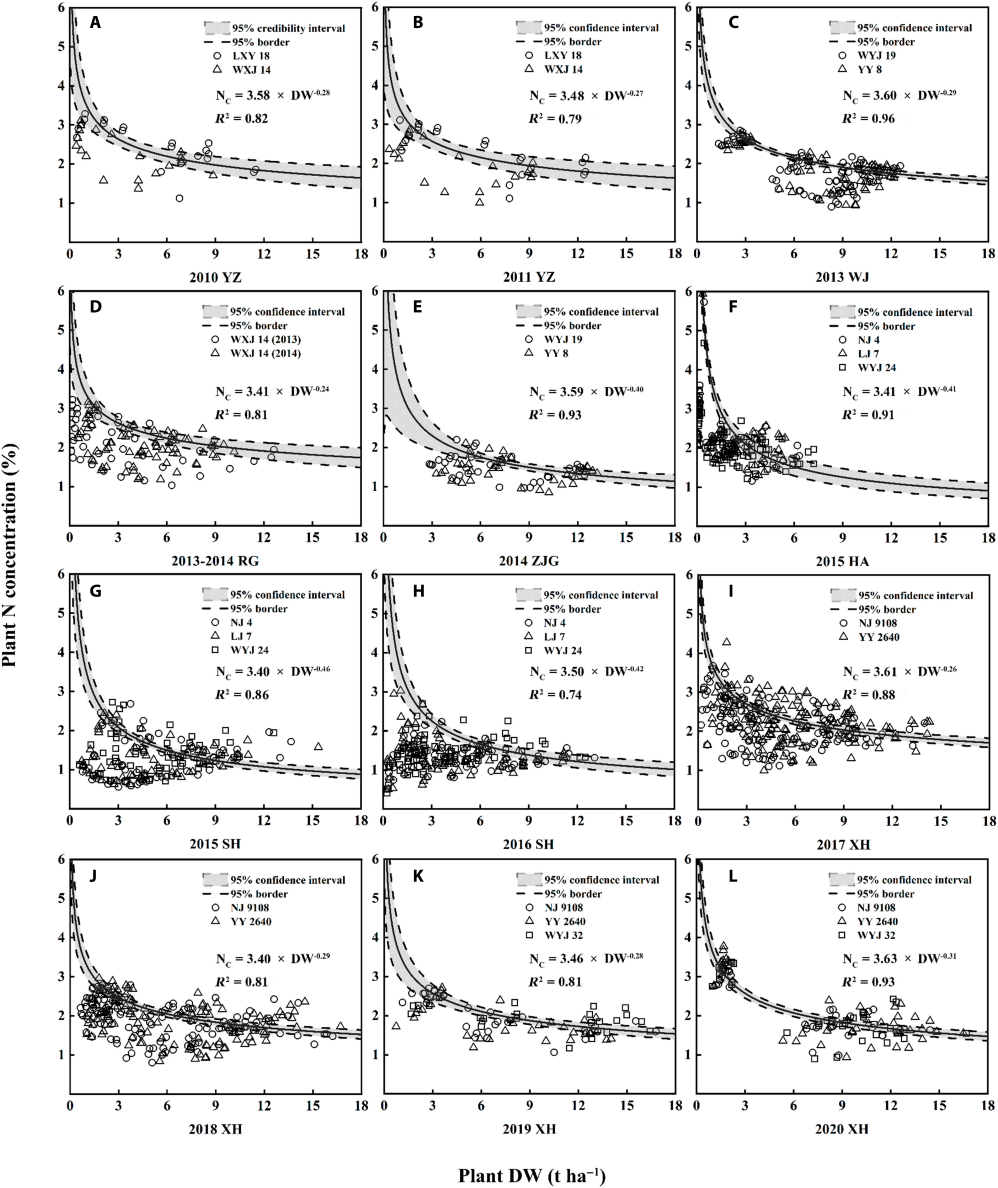
N concentration data points and N_C_ dilution curves obtained from different experiments from 2010 to 2020: Exp. 1 (A), Exp. 2 (B), Exp. 3 (C), Exp. 4 and 5 (D), Exp. 6 (E), Exp. 7 (F), Exp. 8 (G), Exp. 9 (H), Exp. 10 (I), Exp. 11 (J), Exp. 12 (K), and Exp. 13 (L). Gray bands represent 95% confidence interval of curves and dotted lines represent interval border.

As shown in Fig. 2, a total of 12 specific N_C_ dilution curves were established from field experiments. In general, parameter *a* ranged from 3.3958 to 3.6275, and parameter *b* ranged from 0.2390 to 0.4580. There are large differences in the confidence interval widths of curves established by different experiments. The average confidence interval widths of Exp. 1 (Fig. 2A), Exp. 2 (Fig. 2B), and Exp. 6 (Fig. 2E) are 0.56, 0.62, and 0.65, respectively, which are much higher than other experiments (0.25 to 0.48). This is closely related to the sampling times, the fewer sampling times, the greater systematic errors, and the larger confidence width. The confidence width of all curves decreased with the increase in biomass and tended to be stable after the biomass reached 2.5 t ha^−1^. In addition, when plant DW of rice reached 1 t ha^−1^, Japonica rice plants showed similar N concentration due to weak N dilution function at the early growth stage (parameter *a* ranged from 3.40 to 3.63). Furthermore, confidence interval widths under this aboveground biomass are in the range of 0.45 to 1.37, which indicated a high reliability.

### Sources of variation in N_C_ dilution curves

**Fig. 3.**
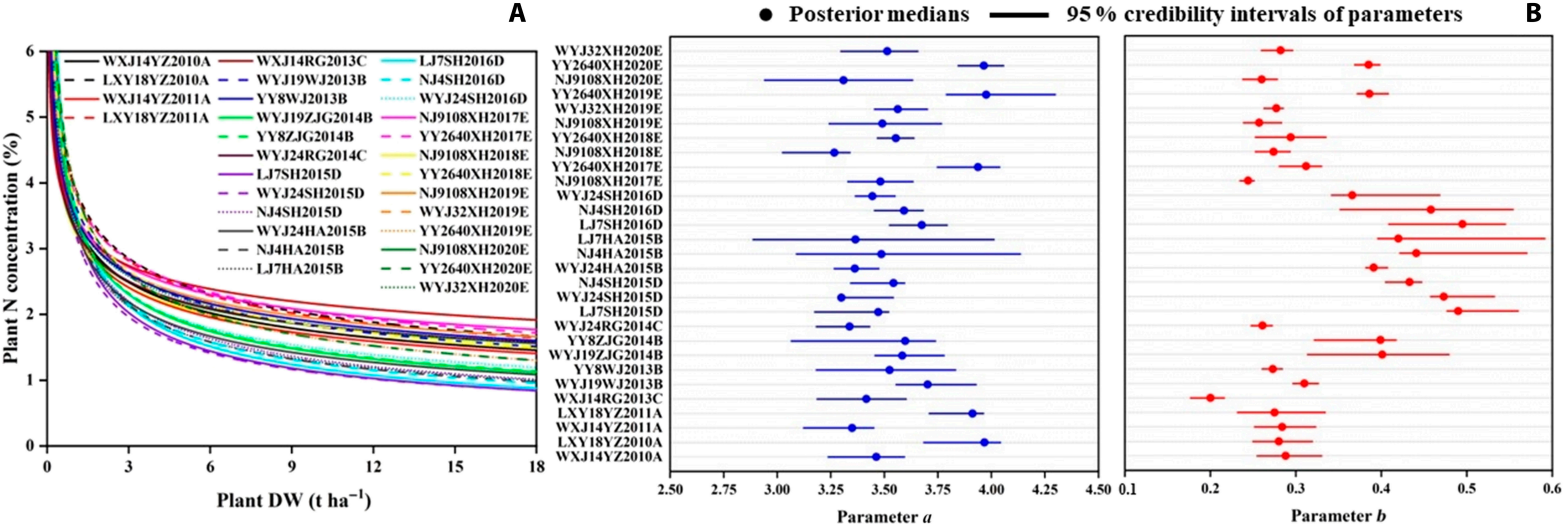
Establishment of specific N_C_ dilution curves under different conditions [each curve represents the median value of the posterior distribution (A)], posterior medians, and 95% credibility intervals of parameters (B).

As shown in Fig. 3, a total of 29 specific N_C_ dilution curves were established based on the BHM and MCMC methods. Parameters *a* and *b* varied with different genetic and environmental conditions (corresponding to cultivar, year, region, and crop management in actual cultivation), resulting in differences between N_C_ dilution curves. Different cultivars (e.g., WXJ14YZ2010A and LXY18YZ2010A, WXJ14YZ2011A and LXY18YZ2011A, and WYJ19WJ2013B and YY8WJ2013B), years (e.g., WXJ14YZ2010A and WXJ14YZ2011A, LXY18YZ2010A and LXY18YZ2011A, and NJ9108XH2017E and NJ9108XH2018E), and region × crop managements (e.g., WYJ24SH2015D and WYJ24HA2015B, NJ4SH2015D and NJ4HA2015B, and LJ7SH2015D and LJ7HA2015B) all had important influences on parameters *a* and *b*. The median values of the posterior distribution were adopted to represent the trend of the specific N_C_ dilution curves, and the descriptive statistics of parameters *a* and *b* are shown in Table 2. The values of parameter *a* ranged from 3.26 to 3.98, while those of parameter *b* ranged from 0.20 to 0.50. The CV for parameter *b* reached 23.82%, indicating that values fluctuate greatly due to various environmental conditions.

**Table 2.** Descriptive statistics of parameters *a* and *b* (the median values obtained from the posterior distribution)

Parameter	Min	Max	CV	Average	SD	Cultivar	Year	Region	Crop management
***a***	3.27	3.98	0.06	3.56	0.21	a	a	a	a
***b***	0.20	0.50	0.24	0.34	0.08	a	a	a	a

^a^Correlation significant at the 0.05 level.

Min, the minimum value; Max, the maximum value; CV, coefficient of variation; SD, standard deviation.

**Fig. 4.**
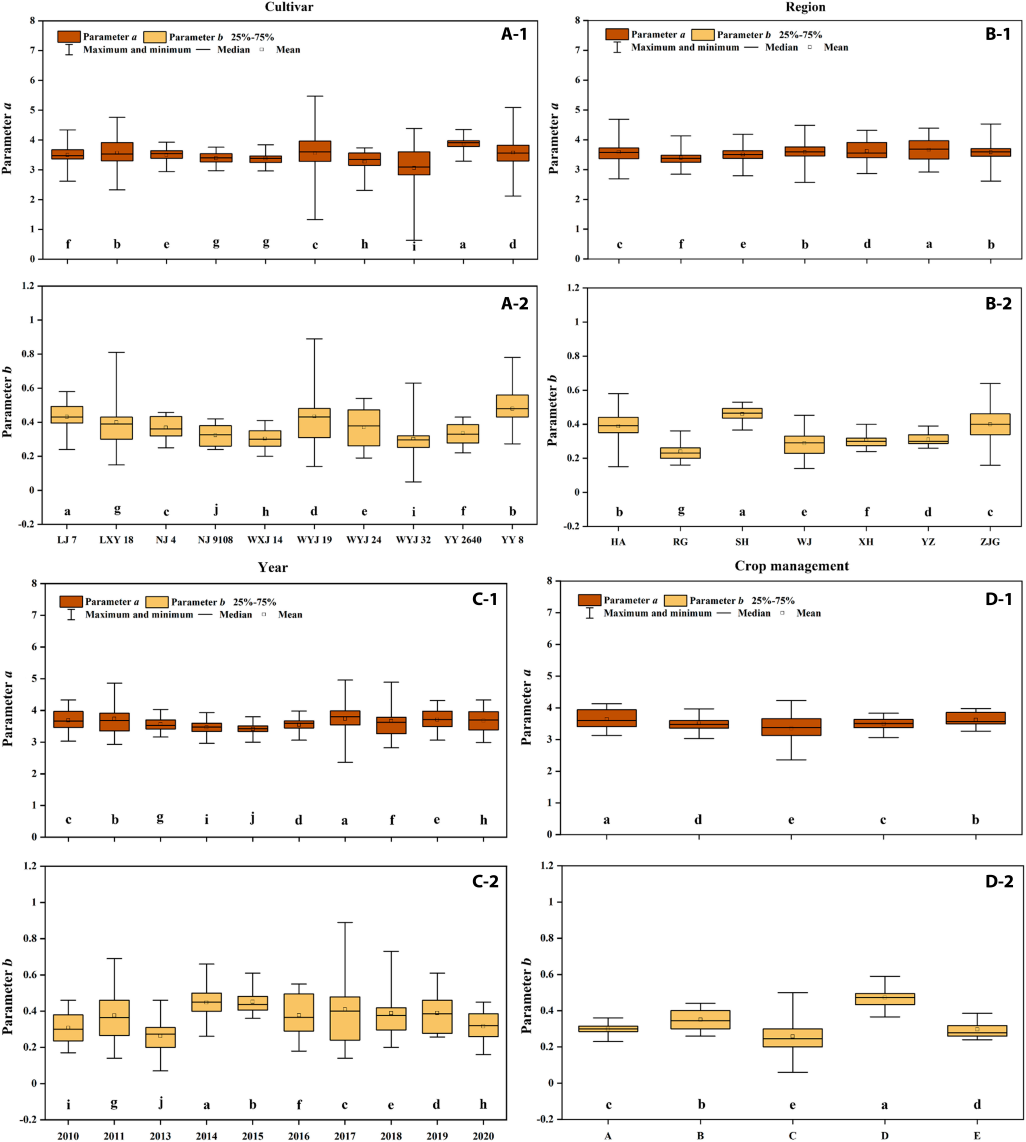
The posterior distribution of parameters *a* and *b* in N_C_ dilution curves under different cultivars (A-1, A-2), regions (B-1, B-2), years (C-1, C-2), and crop managements (D-1, D-2). The different lowercase letters (a, b, c, d, e, f, g, h, i, and j) denote that there was significant difference among treatments at *P* < 0.05.

Considering the actual cultivation environment, the impact of genetic and environmental conditions on parameters *a* and *b* was analyzed from cultivar, region, year, and crop management, respectively (Fig. 4). There were significant differences (*p* < 0.05) in parameters *a* and *b* among different cultivars (except parameter *a* in NJ 9108 and WXJ 14, Fig. 4A-1 and A-2), regions (Fig. 4B-1 and B-2), years (Fig. 4C-1 and C-2), and crop managements (Fig. 4D-1 and D-2). On the whole, the fluctuation range of parameter *a* was 53.78% smaller than that of parameter *b* on average. Parameter *b* was more strongly influenced by the comprehensive effects of genetic and environmental conditions. Parameter *a* changed synchronously with parameter *b*, and the large fluctuation range of parameter *a* was accompanied by the large fluctuation range of parameter *b* (e.g., cultivar-WYJ 19, region-ZJG, year-2017, and crop management-C). For parameter *a*, the distribution range of median values calculated from the posterior distribution between different cultivars, regions, years, and crop managements was 3.06 to 3.87, 3.40 to 3.67, 3.42 to 3.74, and 3.36 to 3.64, respectively. The performance of parameter *a* was relatively stable among different regions, years, and crop managements, while the cultivar had a great influence on parameter *a*. In addition, the median values of parameter *b* were at a range of 0.30 to 0.43, 0.24 to 0.46, 0.26 to 0.45, and 0.26 to 0.47 on different cultivars, regions, years, and crop managements, respectively. Although the median fluctuation range of parameter *b* was relatively smaller among different cultivars, it still fluctuated sharply (Fig. 4A-2).

### Parameter estimation of specific N_C_ dilution curves using RFA and BHM

**Fig. 5.**
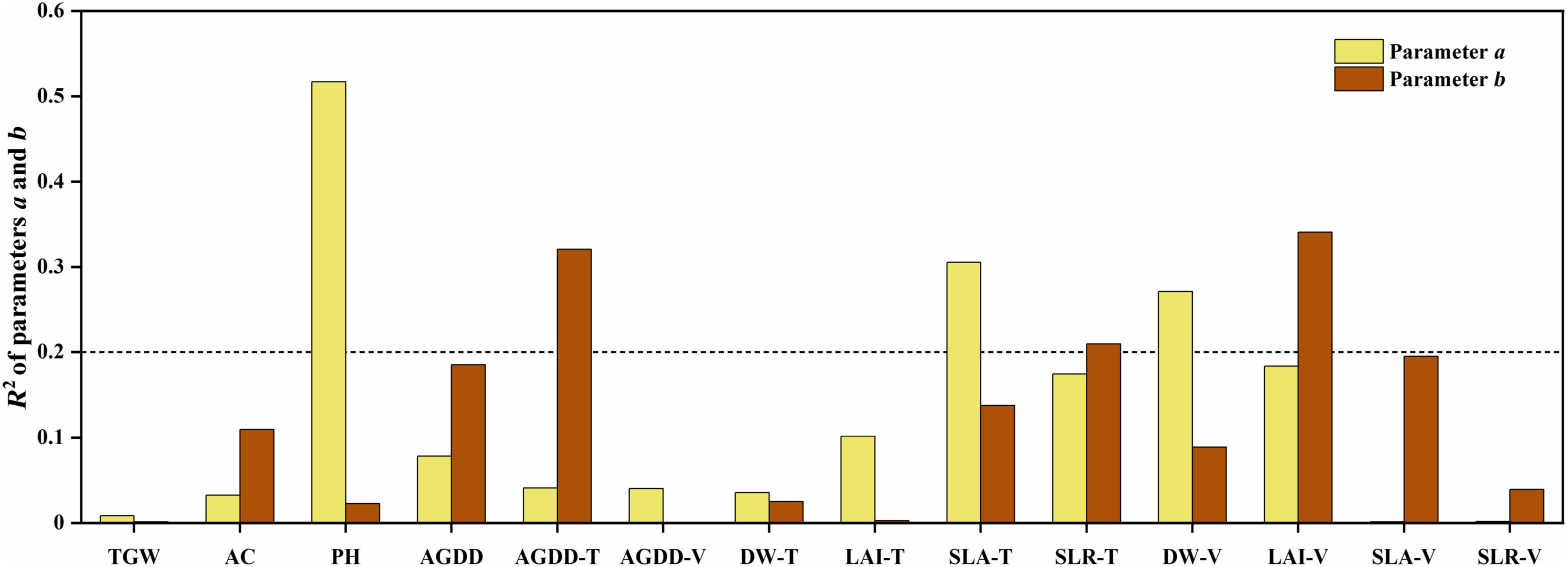
Coefficient of determination (*R*^2^) between indicators and parameters.

The N_C_ dilution curve is influenced by genetic and environmental conditions, which include cultivar, region, year, and crop management in actual cultivation. Therefore, influencing factors from cultivar (*TGW*, *AC*, *PH*, *SLA-T*, and *SLA-V*), year, region, and crop management (*AGDD*, *AGDD-T*, *AGDD-V*, *DW-T*, *LAI-T*, *SLR-T*, *DW-V*, *LAI-V*, and *SLR-V*) were selected respectively for importance analysis (Fig. 5). Coefficient of determination (*R*^2^) varied with different factors and parameters, where the 3 factors most related to parameter *a* were *PH*, *SLA-T*, and *DW-V*, and the 3 factors of largest correlation with parameter *b* were *AGDD-T*, *SLR-T*, and *LAI-V*. The correlation between *TGW* and parameters *a* and *b* was poor, while *SLA-T*, *SLR-T*, and *LAI-V* achieved good performance. In addition, *PH*, *SLA-T*, and *DW-V* predicted parameter *a* better than *b*, while *AGDD-T*, *LAI-V*, and *SLA-V* were more suitable for paramter *b* estimation.

The 3 highly related factors of parameters *a* (*PH*, *SLA-T*, and *DW-V*) and *b* (*AGDD-T*, *SLR-T*, and *LAI-V*) were selected as predictors for estimation, respectively, and leave-one-out cross-validation was adopted for subsequent NNI verification. The RFA method had more advantages than the MLR method in predicting parameters *a* and *b* (Table 3). For parameter *a*, the RFA method was higher than the MLR method 6.58% and 3.64% in modeling *R*^2^ and validation *R*^2^, and lower than 3.46% in RRMSE value. In addition, the RFA method was also superior to the MLR method in modeling *R*^2^, verification *R*^2^, and RRMSE of predicting parameter *b* by 25.78%, 11.30%, and 4.28%, respectively.

**Table 3.** Comparison of different modeling methods for estimating parameters *a* and *b*

Parameters	Modeling methods	Modeling *R*^2^	Validation *R*^2^	Validation RRMSE
***a***	**MLR**	0.60	0.65	3.47%
	**RFA (NTREE = 500, MTRY = 3)**	0.64	0.67	3.35%
***b***	**MLR**	0.44	0.50	16.83%
	**RFA (NTREE = 500, MTRY = 4)**	0.55	0.56	16.11%

**Fig. 6.**
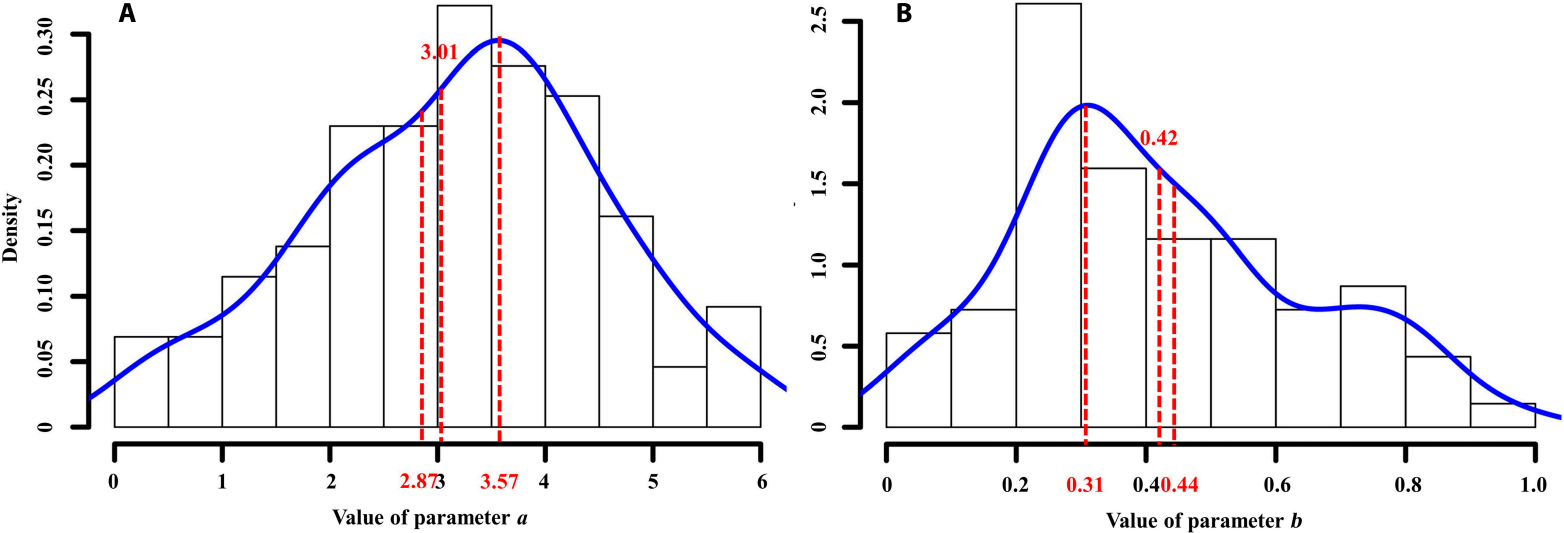
Posterior distributions of parameters *a* (A) and *b* (B) for N_C_ dilution curve. Density indicates the frequency within a certain interval.

Figure 6 shows parameter posterior distributions through the BHM and MCMC methods, and the results were presented in the form of a frequency distribution histogram and kernel density function fitting curve. Parameters *a* and *b* of the universal N_C_ dilution curve were determined by *Mean* (*a* = 2.87, *b* = 0.44), *Median* (*a* = 3.01, *b* = 0.42), and *MPN* (*a* = 3.57, *b* = 0.31) from kernel density function fitting curves, respectively (Table 4).

**Table 4.** Determination of parameters *a* and *b* in universal N_C_ dilution curves.

Parameters	SDM	Average	RFA	BHM		
				Mean	Median	MPN
***a***	3.36	3.56	3.56	2.87	3.01	3.57
***b***	0.30	0.34	0.34	0.44	0.42	0.31

*SDM*, parameter estimation based on simple data-mixing; *Average*, parameter estimation through averaging specific curve parameters; *RFA*, parameter estimation through averaging predicted specific curve parameters; *BHM* (*Mean*, *Median*, and *MPN*), parameter estimation through selecting representative values (*Mean*, *Median*, and *most probable number*) from posterior distributions based on the Bayesian hierarchical model.

### The NNI validations of the established universal N_C_ dilution curves

**Fig. 7.**
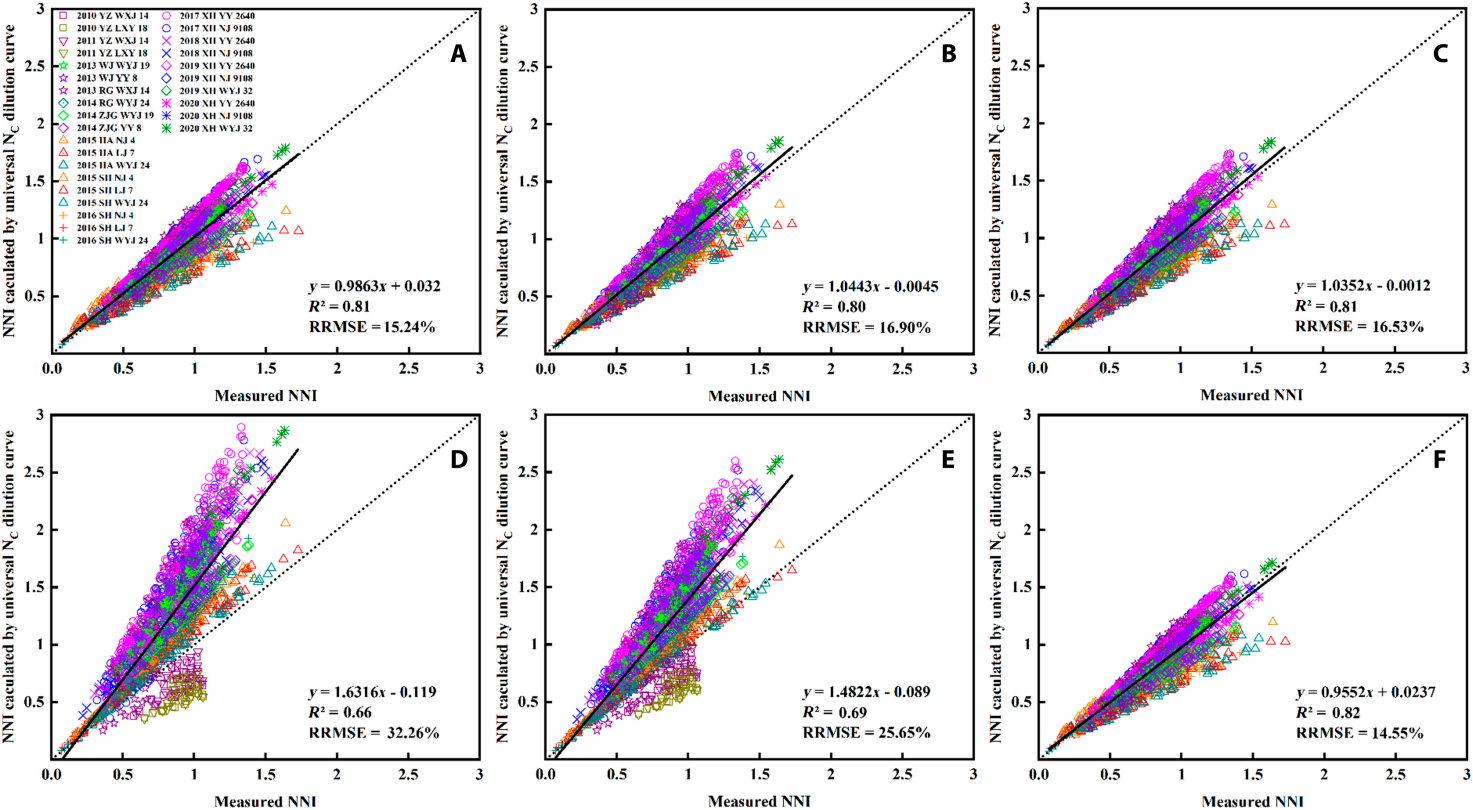
NNI validation results of universal N_C_ dilution curves based on *SDM* (A), *Average* (B), *RFA* (C), *Mean* (D), *Median* (E), and *MPN* (F). The measured NNI values were calculated by each specific N_C_ dilution curve based on the traditional method.

The NNI value calculated by each specific N_C_ dilution curve based on the traditional method was taken as the reference value (measured value). As shown in Fig. 7, the NNI verified *R*^2^ of *MPN* reached 0.82, while *Mean* and *Median* had a poor ability of N diagnosis. There were 2 other approaches (RFA and SDM) for establishing the universal N_C_ dilution curve (Table 4), and the corresponding validation results of NNI are shown in Fig. 7. The RFA approach aimed to determine parameters *a* and *b* by averaging the estimated parameter values from the specific curves above (Table 4, *RFA*, *a* = 3.56, *b* = 0.34) with an NNI validation *R*^2^ of 0.81 (Fig. 7C). In addition, the average value of specific N_C_ dilution curves also showed a good validation result of NNI (Table 4, *Average*, *a* = 3.56, *b* = 0.34, Fig. 7B, *R*^2^ = 0.80). The SDM approach aimed to fit all the N_C_ points and build the mixed curve (Table 4, *SDM*, *a* = 3.36, *b* = 0.30), which obtained a high NNI validation *R*^2^ of 0.8138 (Fig. 7A).

In general, there was no significant statistical difference of NNI validations between the universal N_C_ dilution curves based on *SDM*, *Average*, *RFA*, and *MPN*. However, *Mean* and *Median* were far less capable of N diagnosis than *MPN*. Results indicated that universal N_C_ dilution curves established by *SDM*, *Average*, *RFA*, and *MPN* had strong applicability and robustness.

## Discussion

### Uncertainty analysis of parameters *a* and *b*

Fewer data points and more outliers resulted in higher confidence interval and uncertainty of specific N_C_ dilution curve in each experiment, which is due to the lower sampling frequency (e.g., Exp. 1, Exp. 2, and Exp. 6). This can also well explain the N_C_ dilution curve in Exp. 6, which had a large confidence width because of no sampling points before plant DW reached 3 t ha^−1^. A smaller sample number also resulted in a larger posterior distribution of parameters *a* and *b* based on the BHM approach (e.g., cultivar-WYJ 19, region-ZJG, and year-2017). At about the tillering end of rice, the plant DW reaches 1 t ha^−1^, and the N concentration at this time represents the value of parameter *a*. This is an important stage closely related to the value of parameter *a*, which is also the reason for using this stage to construct parameter predictors (e.g., AGDD-T, DW-T, LAI-T, SLR-T, and SLA-T). Parameter *a* can reflect the N absorption characteristics of rice plants, which is mainly determined by cultivars. This study aimed at different Japonica rice cultivars; thus, the fluctuation range of parameter *a* was relatively stable even under different environmental conditions. Compared with region, year, and crop management, parameters *a* and *b* both had a smaller fluctuation range among different cultivars. There were many factors affecting parameter *b* a lot, which made it difficult to predict it accurately. *AGDD-T*, *SLR-T*, and *LAI-V* were the factors highly related to parameter *b*. Plant N concentration dilution is caused by the physiological process of crop growth and development. Plants grow higher to obtain more sunlight, leading to the change of dry matter distribution, which is greatly associated with parameter *b* [38] and reflects on *SLR*. Moreover, the shading phenomenon between leaves becomes more obvious with the progress of crop growth. In order to make effective use of N nutrition in crop canopy, N in shaded leaves was transported to the top leaves, resulting in the decrease of leaf N concentration. This process is closely related to *LAI*, which is an important indicator of parameter *b*. *AGDD-T* indicated that parameter *b* was affected by growth days and temperature. As another characterization of crop growth status other than *LAI*, *DW* (*DW-V*) also had a great impact on parameter *a*. Nevertheless, rice cultivar characteristics affected parameter *a* a lot, and *PH* and *SLA-T* are both such indicators. Moreover, *DW-V* represents the change in crop biomass influenced by various factors. There was no temperature-related factor that showed great impact on parameter *a*, indicating that the environmental conditions’ experimental sites were similar and relatively stable at the early growth stage of Japonica rice.

### Parameter estimation of specific N_C_ curves

This study provided a new approach for predicting parameters a and b from specific N_C_ dilution curves, while the unity of predictors still needs to be verified in the future due to the limited experimental data. In particular, PH showed a very high contribution to parameter a estimation, which may be caused by a limited dataset. However, the reference values (TGW, AC, and PH) from the China Rice Data Center were empirical with strong uncertainty. Although only PH was used in the prediction of parameter a in this study, more reliable cultivar indicators need to be explored. For the selection of indices, there was still a lot of room to explore (e.g., soil indicators and management measurements). Dry matter accumulation and N concentration changes of japonica rice involve complex physiological processes, and simple linear regression models are difficult to deal with such nonlinear problems. Nevertheless, this issue can be better handled by the machine learning method (RFA), which can be compatible with input samples from various dimensions, and also achieve accurate prediction in the case of lacking value [39]. RFA has been widely used in crop yield prediction and other aspects [40,41]. In order to enhance the applicability of parameter estimation in specific N_C_ dilution curves, only highly related factors were selected as predictors. In fact, factors affecting the value of parameter a and b are different, and relatively stable factors with higher importance are expected to be found in the future. In addition, the parameter tuning process can be carried out on the RFA method in this study, which may further improve the prediction accuracy [42]. Furthermore, more machine learning methods (e.g., artificial neural network and support vector machine) should also be taken into consideration to compare and explore better solutions [43,44].

### The establishment of universal N_C_ dilution curves

It has always been a hot issue to establish a reliable universal N_C_ dilution curve with strong applicability [45]. This study explored 3 feasible approaches for Japonica rice in Yangtze River Reaches. SDM, as a traditional approach, achieves a high verification accuracy of NNI through fitting all the N_C_ points. However, the determination of N_C_ point and subsequent data processing are undoubtedly time-consuming [46], which is not conducive to the actual production application. Therefore, simplifying the complex steps while ensuring N diagnostic capacity is of great significance. In this study, results showed that universal N_C_ dilution curves determined by averaging parameters a and b from specific curves also had good N diagnostic ability, which is consistent with Yao et al. [24]. Moreover, on the basis of predicting specific N_C_ dilution curve parameters with highly related indicators, it can be extended to the rapid estimation of universal curves by averaging parameters a and b. This approach can greatly improve modeling efficiency, but more practice is needed in the selection of predictors. In addition, the BHM for N_C_ dilution curves proposed by Makowski et al. [17] can also skip the step of distinguishing N-limiting and non-N-limiting groups. Parameter posterior distributions can be easily obtained from basic data and prior knowledge through BHM and MCMC methods. Previous research always adopted the MCMC method for uncertainty analysis of model parameters, while few focused on parameter estimation and verification [47,48]. According to the posterior distribution of parameters a and b, 3 representative values (Mean, Median, and MPN) were selected to explore the potential of establishing universal N_C_ dilution curves. Although MPN achieved excellent results of NNI validation, there were still some uncertainties. On one hand, prior knowledge almost directly determined the upper and lower limits of parameter posterior distributions. On the other hand, selected representative values may not fully represent the actual values, even if they have been applied with high precision. The applicability of MPN was proved in this study, and its stability is expected to be verified in the future.

In this study, the sources of the differences among specific N_C_ dilution curves were from cultivars, years, regions, and crop managements. For Japonica rice, parameter *a* is mainly determined by the N absorption characteristics of cultivars, while parameter *b* is related to cultivars and environmental conditions (e.g., AGDD and rain–heat resources). Highly related indicators (*PH*, *SLA-T*, *DW-V*, *AGDD-T*, *SLR-T*, and *LAI-V*) were selected from 14 factors to explore the potential of predicting parameters *a* and *b*. In the parameter estimation of specific N_C_ dilution curves, the RFA method was, on average, 14.69% and 6.98% higher than the MLR method in the accuracy of modeling and verification, respectively. In addition, *SDM*, *Average*, *RFA*, *Mean*, *Median*, and *MPN* determined parameters of the universal N_C_ dilution curves for Japonica Rice in Yangtze River Reaches, among which, *SDM*, *Average*, *RFA,* and *MPN* obtained a good performance with NNI validation *R*^2^ above 0.80. The good validation accuracy of *Average* provided theoretical support for the implementation of the *RFA* approach. Compared with the *SDM* approach, *RFA* and *MPN* approaches simplified complex steps, improved operational efficiency, and showed strong applicability.

## Data Availability

The data used to support the findings of this study are included within the article.
